# Vitamin D-Related Genetics as Predictive Biomarker of Clinical Remission in Adalimumab-Treated Patients Affected by Crohn’s Disease: A Pilot Study

**DOI:** 10.3390/ph14121230

**Published:** 2021-11-27

**Authors:** Jessica Cusato, Lorenzo Bertani, Miriam Antonucci, Cristina Tomasello, Gian Paolo Caviglia, Simone Dibitetto, Alessandro Massano, Michela Mangia, Jacopo Mula, Linda Ceccarelli, Francesco Costa, Federico Zanzi, Marco Astegiano, Davide Giuseppe Ribaldone, Antonio D’Avolio

**Affiliations:** 1 Laboratory of Clinical Pharmacology and Pharmacogenetics, Department of Medical Sciences, University of Turin, Amedeo di Savoia Hospital, Corso Svizzera, 164, 10149 Turin, Italy; miriam.antonucci20@gmail.com (M.A.); jacopo.mula@unito.it (J.M.); antonio.davolio@unito.it (A.D.); 2 Department of Translational Research and New Technologies in Medicine and Surgery, University of Pisa, 56126 Pisa, Italy; lorenzobertani@gmail.com (L.B.); federico.zanzi@outlook.com (F.Z.); 3 S.C. Farmacie Ospedaliere-Ospedale M.Vittoria-ASL Città di Torino, 10144 Turin, Italy; cristina.tomasello@aslcittaditorino.it; 4 Unit of Gastroenterology, Department of Medical Sciences, University of Turin, 10124 Turin, Italy; simone.dibitetto@edu.unito.it (S.D.); alessandro.massano@unito.it (A.M.); mickela.89.mm@gmail.com (M.M.); davidegiuseppe.ribaldone@unito.it (D.G.R.); 5 IBD Unit, Department of General Surgery and Gastroenterology, Pisa University Hospital, 56124 Pisa, Italy; ceccarellilinda@gmail.com (L.C.); fcosta@med.unipi.it (F.C.); 6 Unit of Gastroenterology, Molinette Hospital, 10126 Turin, Italy; marcoastegiano58@gmail.com

**Keywords:** *VDR*, *CYP27B1*, *GC*, SNPs, personalized medicine

## Abstract

Adalimumab (ADA) is a human anti-tumor necrosis factor (TNF-α) monoclonal antibody used in inflammatory bowel diseases, such as Crohn’s disease (CD). Vitamin-D (VD) is important for biological functions, such as the modulation of expression of genes encoding enzymes and transporters involved in drug metabolism and transport. ADA trough levels were associated with VD concentrations in patients with IBD, but no data are present in the literature concerning VD pathway-related gene single-nucleotide polymorphisms (SNPs) in affecting clinical outcomes. For this reason, the aim of this study was to evaluate the ability of VD-related genetics to predict clinical remission at 3 and 12 months in patients affected by CD treated with ADA. Patients affected by CD were included in this study. SNPs in *CYP27B1*, *CYP24A1*, *GC*, and *VDR* genes were analyzed through real-time PCR. A total of 63 patients were enrolled. Calprotectin, hemoglobin, and C-reactive protein levels were influenced by SNPs in *VDR*, *CYP27B1*, and *GC* genes. After 3 months of therapy, clinical remission was predicted by smoke, systemic steroids, and *VDR* BsmI, whereas at 12 months by *GC* 1296AA/AC and VD supplementation. This study reports the association between VD pathway-related genetics and ADA treatment. Further studies are needed to confirm these promising data.

## 1. Introduction

Inflammatory bowel diseases (IBD), including Crohn’s disease (CD) and ulcerative colitis (UC), are multifactorial, lifetime, inflammatory diseases of the gastrointestinal tract, with an increasing prevalence worldwide [1]. There is not a definitive cure, but many new drugs (biologics and small molecules) have been approved in recent years demonstrated to improve the disease course and quality of life of these patients [2]. Unfortunately, the clinical remission rate obtained with these drugs is only about 30%, with a significant amount of costs and side effects that could be avoided if we could personalize therapy, identifying the right drug for the right patient [3]. From this perspective, several biomarkers have been proposed in order to predict therapeutic effectiveness [4,5,6,7,8,9], but their use in clinical practice is still limited, since an approach with a single biomarker presents certain concerns [10]. Therefore, the future of inflammatory bowel disease research is moving through the identification of the greatest number of putative biomarkers, in order to develop a model integrating serum or stool biomarkers with pharmacogenetics [11].

Worldwide, vitamin D (VD) deficiency represents a public health problem in all age groups, although studies are still lacking in most countries, particularly at -risk groups. The National Health and Nutrition Examination Survey (NHANES) shows that 79% of the elderly adult population have VD deficiency or insufficiency [12,13]. This phenomenon seems to be frequent in Italy among elderly adults, particularly during winter months [14,15]. Different diseases have been associated with VD deficiency, including COVID-19 [16].

Many studies displayed a correlation between VD reduced plasma levels and the onset of bone, neurological, cardiovascular diseases, diabetes, metabolic syndrome, infections, cognitive decline, and neurodegenerative diseases and neoplasms and autoimmune disorders [17,18,19,20]. In addition, VD plasma deficiency levels seem to be associated with liver disease progression in the response to interferon-based treatment in HCV-related hepatopathies [21]. 

Ultraviolet light is responsible for VD synthesis in the skin; cholecalciferol is hydroxylated to calcifediol (25-hydroxy VD, 25-VD) in the liver through cytochrome P-450 (CYP) 27A1 and CYP2R1 and, in the kidney, calcitriol (1,25-dihydroxy VD, 1,25-VD) is synthesized through CYP27B1. 1,25-VD is then transported in the bloodstream through the vitamin D binding protein (VDBP). Cholecalciferol is inactivated to calcitroic acid by the CYP24A1 enzyme, whose expression is partially affected by calcium and parathyroid blood concentrations and partially induced by 1,25-VD, which can also directly down-regulate the CYP27B1 gene. 

Through its receptor VDR, VD modulates the expression of genes encoding transporters and enzymes responsible for drug transport and metabolism, such as cytochromes (CYPs) [22]. Drocourt et al. showed that, through electrophoretic mobility shift assays and cotransfection in HepG2 cells using wild-type and mutated oligonucleotides, VD upregulates *CYP3A4* and, to a lesser extent, *CYP2B6* and *CYP2C9* genes expression in human hepatocytes. In fact, they revealed that VDR recognizes and transactivates *CYP3A4*, *CYP2B6*, and *CYP2C9* promoters binding to xenobiotic-responsive elements; in addition, it is targeted by the constitutive androstane receptor (CAR) and/or the pregnane X receptor (PXR). Moreover, the authors suggested that VDR, PXR, and CAR modulate several CYP genes’ expression by competitively interacting with the same battery of responsive elements [23].

Several drugs are metabolized by CYP3A4; this gene shows VD responsive elements (VDRE) and its expression is upregulated in presence of 1,25-VD. Lindh et al. suggested tacrolimus and sirolimus seasonal variability according to changes in VD levels, with lower drug concentrations in July to September than in January to March. Furthermore, VD influence on CYP3A4 expression results in altered metabolized drugs concentration as shown for immunosuppressants [24,25,26].

VD may interact with several drugs, potentially altering drug toxicity or efficacy, but drugs may also affect VD metabolism and status [27]: The 25-hydroxylase CYP3A4 is able to convert precursors to 25-VD, whereas several in vitro studies showed that the anti-HIV drug ritonavir inhibits 1,25-VD degradation and 25-VD to 1,25-VD conversion [28,29]. Moreover, antiretroviral drugs are PXR ligands, thus able to activate it and the related pathway [30]: PXR is involved in xenobiotics and drugs detoxifications, and in fact, it binds to VDRE, upregulating 24-hydroxylases and other CYPs related to the VD pathway. 

VD plays an important role in IBD during anti-tumor necrosis factor (TNF) treatment: Higher rates of clinical response and clinical remission correlate with increased VD levels [31]. Santos-Antunes et al. seem to partially explain this finding with VD–immune system interaction. The authors also showed that higher rates of anti-TNF failure and adverse events are in association with increased levels of anti-nuclear antibodies in patients with VD deficiency [32,33]. Moreover, serum trough levels of Infliximab (IFX) and Adalimumab (ADA) seem to be associated with VD levels in patients with IBD [34]. 

An association between vitamin D pathway-related gene single-nucleotide polymorphisms (SNPs) and ADA clinical outcome, from the perspective of a putative use as a biomarker of the therapeutic response, has never been assessed. For this reason, the aim of this study was to evaluate the ability of VD-related genetics to predict clinical remission in patients affected by CD treated with ADA, for the first time in the literature.

## 2. Results

A total of 63 patients were enrolled in Turin and Pisa; their characteristics are reported in Table 1. There were 36 males (57.1%) and the median age was 42 years (IQR 32;55), whereas the median years of illness were 13 (IQR 6.5;18.5). Thirty-one (49.2%) subjects were supplemented with VD and clinical remission at 3 and 12 months was achieved in 37 (58.7%) and 36 (57.1%) patients, respectively.

Subsequently, patients’ clinical characteristics were analyzed according to SNPs of genes related to VD metabolism, transport, and activity.

### 2.1. Calprotectin

**Figure 1:**
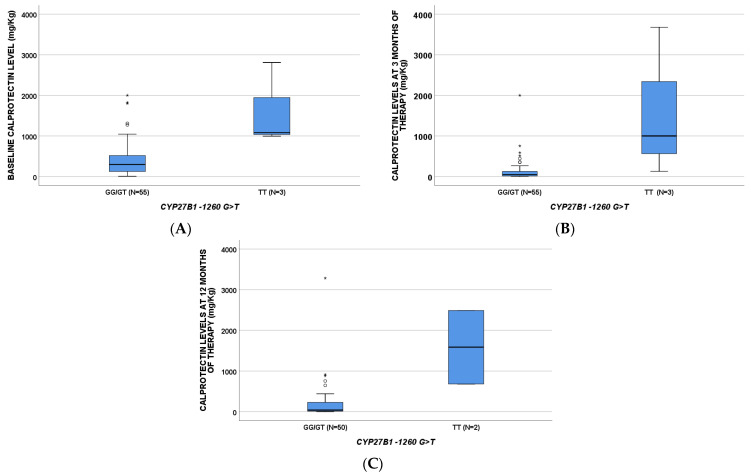
Calprotectin levels according to *CYP27B1*-1260G>T SNP at baseline (**A**, *p* = 0.018), three (**B**, *p* = 0.015), and twelve (**C**, *p* = 0.018) months of therapy. *p*-values are obtained through Mann–Whitney test. * Circles and stars indicate “out” values (small circle) and “far out” values (star).

The levels of calprotectin were different according to the genetic variants (Table 2); particularly, *CYP27B1*-1260 TT was able to affect all three time points (Figure 1). At BL, *CYP27B1* + 2838 CT/TT (*p =* 0.010), *VDR* ApaI CA/AA (*p* = 0.021), *VDR* Cdx2 AG/GG (*p* = 0.036), and *GC* 1296 CC (*p* = 0.034) had an influence; finally, a role was suggested for *CYP27B1* + 2838 CT/TT (*p* = 0.023), *VDR* ApaI CA/AA (*p* = 0.021), and *VDR* TaqI TC/CC (*p* = 0.046) at 3 months of therapy. 

### 2.2. Hemoglobin

**Figure 2:**
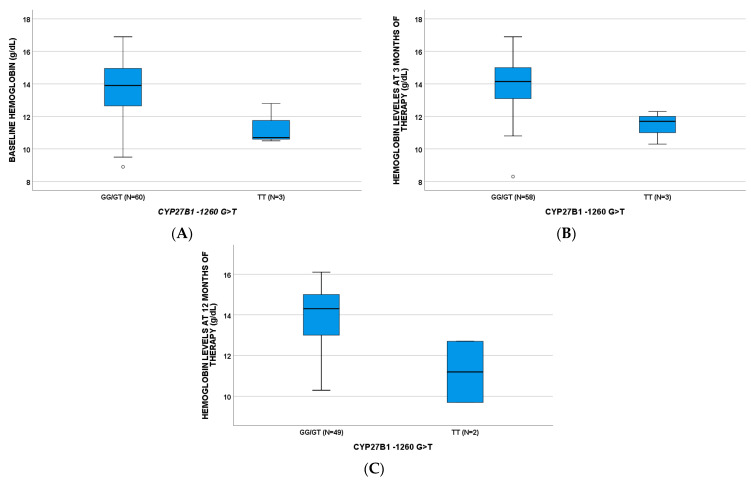
Hemoglobin levels according to *CYP27B1*-1260G>T SNP at baseline (**A**, BL, *p* = 0.023), three (**B**, *p* = 0.011), and twelve (**C**, *p* = 0.047) months of therapy. *p*-values are obtained through Mann–Whitney test.

Hemoglobin (Hb) levels were influenced by genetic polymorphisms (Table 2); also, in this case, *CYP27B1*-1260 TT was able to affect the three analyzed time points (Figure 2). *CYP24A1* 8620 GG plays a role at BL and 3 months with *p*-values of 0.041 and 0.034, respectively; *GC* 1296 CC influenced Hb concentrations at 12 months of treatment (*p* = 0.046). 

### 2.3. C-Reactive Protein > 1 mg/dL

The C-reactive protein (CRP) was affected by *VDR* TaqI CC (*p* = 0.036) at *BL, CYP27B1*-1260 TT (*p* = 0.017) at 3 months, and, finally, VDR ApaI AA (*p* = 0.037) at 12 months.

**Figure 3:**
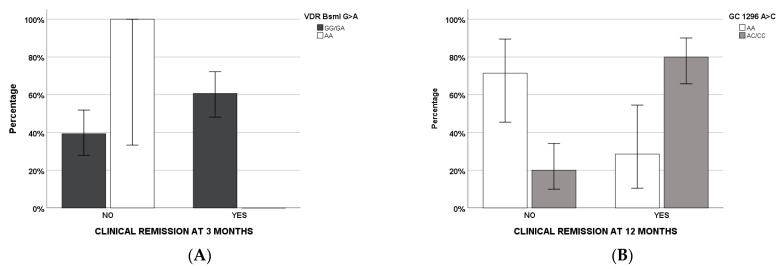
Clinical remissions according to genetic variants (*VDR* BsmI G>A in (**A**), *GC* 1296 A>C in (**B**)) that remained in the final regression models.

Clinical remission at 3 (Table 3) and 12 (Table 4) months of therapy was evaluated through regression analyses (odds ratio and interval of confidence at 95%): Demographical, clinical, pharmacological, and genetic factors were considered firstly in the univariate and, subsequently, in the multivariate models. For the 3-month timepoint, smoke, systemic steroids, and *VDR* BsmI AA remained in the multivariate regression, whereas *GC* 1296 AA/AC and VD supplementation were retained in the final regression model at 12 months of therapy (Figure 3).

## 3. Discussion

VD is an essential fat-soluble vitamin for calcium maintenance homeostasis, bone health, and preventing falls and fractures. Ultraviolet light is responsible for VD synthesis in the skin; in the liver, cytochrome P-450 (CYP)27A1 and CYP2R1 hydroxylate cholecalciferol to calcifediol (25-hydroxy VD, 25-VD); and in the kidney, CYP27B1 synthesizes calcitriol (1,25-dihydroxy VD, 1,25-VD). 1,25-VD is then transported in the bloodstream through the vitamin D binding protein (VDBP) encoded by the *GC* gene. Cholecalciferol is inactivated to calcitroic acid by the CYP24A1 enzyme, whose expression is partially affected by calcium and parathyroid blood concentrations and partially induced by 1,25-VD [30], which can also directly down-regulate the *CYP27B1* gene [17,35]. VD levels are higher in men than in women, but they are lower in people that are older and have darker skin pigmentation [36,37].

VD deficiency is highly prevalent in IBD, involving up to 80% of apparently well-nourished patients [38]. However, VD-related parameters have never been associated with the therapeutic response in IBD patients. Moreover, currently, no strong factors aiming to predict clinical remission at three and twelve months of therapy are used in the routine clinical practice in IBD care.

In this study, 63 patients affected by CD were enrolled and evaluated; particularly, predictive factors of biochemical biomarkers, such as calprotectin, Hb, and CRP, and clinical remission at three and twelve months of therapy were investigated.

We focused on VD-related genetics, since no data are available in literature in this context. Polymorphisms in genes encoding its activation enzyme (CYP27B1), its inactivation enzyme (CYP24A1), its receptor (VDR), and its transporter (VDBP) were analyzed and related to clinical features.

Interestingly, *CYP27B1* -1260 TT was found to be associated with calprotectin and Hb at BL, three, and twelve months of therapy. In particular, this genotype patients showed higher calprotectin levels and lower Hb concentrations. These two findings could be related, since increased calprotectin exposure could be associated with a high grade of inflammation and blood loss, with consequently reduced Hb levels [39,40]. Furthermore, it is important to consider that *CYP27B1* -1260 TT was associated with increased colorectal cancer risk; the *CYP27B1* gene encodes a member of the CYP monooxygenases family (the 1α-hydroxylase enzyme), localized to the inner mitochondrial membrane of renal cells [41]. Available data on the *CYP27B1* -1260 rs10877012 promoter polymorphism are inconsistent: Lange and colleagues suggested its influence on 1,25-VD serum concentrations, in contrast of what was observed by Kitanaka et al. [42,43,44,45].

For the first time, clinical remissions at three and twelve months after starting therapy were analyzed according to VD-related genetic variants. We suggested that the *VDR* BsmI AA genotype, in addition to smoke and use of systemic steroids, was a predictive factor at three months of therapy, whereas *GC* 1296 AA/AC was at twelve months. In particular, all the factors, with the exception of *GC* 1296 AC/CC, are negative predictors.

The role of *GC* 1296 is unknown: It encodes the VDBP, which is a multifunctional glycoprotein produced by hepatic parenchymal cells and secreted into the circulation [41]. Its functions include VD transport in blood, actin scavenging, and fatty acid binding. It is downregulated in several diseases, such as sepsis, cutaneous malignant melanoma, hepatocellular carcinoma, primary non-metastatic breast cancer, type I diabetes, and patients with IBD [46]. Our recent study suggested that this genotype was associated with the area under the curve efficacy cut-off value of deferasirox-treated subjects, confirming our results suggesting a potential role in predicting a positive clinical response [47].

VDR is the VD receptor that regulates the transcription of several genes; furthermore, it modulates several physiological systems, such as immune, calcium, and phosphorous homeostasis or apoptosis. Several VDR gene SNPs were able to affect certain features in different diseases, for example non-small-cell lung cancer or autoimmune hepatitis^4^^9^. It was supposed that VDR mRNA expression and protein levels could be lower in IBD affected subjects [48]. However, the functional effect of this genetic variant remains unclear: Some studies suggested it could influence *VDR*-mRNA expression, affecting its stability, confirming previous studies [49].

Concerning clinical remission at 12 months of therapy, VD supplementation remained in the final model as a negative predictive factor: It could be possible that these patients have lower VD levels, which could not be normalized, despite the supplementation. This finding is in line with the current literature, which displayed how the vast majority of patients with IBD have low levels of VD, regardless of their clinical or nutrition conditions [38,50].

The present study has several important limitations. Firstly, the number of patients was small; however, it is worth noting that this factor did not affect the significance of the analyses, thus suggesting that the putative associations of VD genetics and clinical outcome of ADA treatment in CD are conceivable. Secondly, data concerning VD exposures would have significantly improved our results, but unfortunately, these data were unknown. Finally, an endoscopic assessment at twelve months would improve the significance of the results, although fecal calprotectin is currently well recognized as a marker of endoscopic remission [5,51].

## 4. Materials and Methods

### 4.1. Study Design

We performed a prospective, multicenter study at the Gastroenterology Unit of “Città della Salute e della Scienza di Torino”, Italy and the IBD Unit, Pisa University Hospital, Pisa, Italy. From January 2018 to January 2020, we recruited consecutive patients: (1) Affected by CD with indications to treatment with ADA; (2) naive to anti-TNF drugs or other biological drugs; (3) older than or equal to 18 years; (4) who agreed to sign the informed consent to participate in the study. We treated patients with moderate-to-severe disease activity or steroid-dependent disease with previous failure or intolerance to thiopurines with ADA. Exclusion criteria were: (1) Refusal to participate to the study; 2) pregnancy; 3) other concomitant auto-immune diseases (such as psoriasis, arthritis, etc.). Clinical history, data on physical examination, recent biochemical examinations, and signed informed consent for the purpose of enrolment in the study were collected. All patients were treated with a total dose of 160 mg at day 1, 80 mg at day 15, and 40 mg of ADA by subcutaneous administration every two weeks. Before starting ADA therapy, 9 mL of venous blood was collected. The blood samples were associated with an anonymous numerical identification code and stored frozen at −80 °C. In a subgroup of patients, randomly identified, a second blood sample was collected after 3 months of treatment (T1).

Clinical characteristics were evaluated at BL (T0, before starting treatment), 3, and 12 months of therapy. At the same time-points, fecal calprotectin was assessed by ELISA (Calprest^®^, Trieste, Italy).

The primary outcome was the prediction of clinical remission at 3 and 12 months of ADA therapy (T3 and T12). Clinical response to ADA therapy was defined as a decrease in the Harvey–Bradshaw index (HBI) score used to foster a systematic collection of clinical data related to Crohn’s disease: It considers five parameters, exclusively clinical (patient well-being, abdominal pain, number of liquid or soft stools, abdominal mass, and complications). For each parameter, a specific score is assigned: Greater than or equal to 3 in the absence of corticosteroid therapy and with ongoing ADA therapy, clinical remission, and HBI <= 4, in agreement with the literature [52]. The study followed the principles of the Declaration of Helsinki and was approved by the local ethical committees: Comitato Etico Interaziendale A.O.U. Città della Salute e della Scienza di Torino—A.O. Ordine Mauriziano—A.S.L. Città di Torino (approval code 0056924); Comitato Etico Regionale Toscana Area Vasta Nord Ovest—CEAVNO (approval code 16790).

### 4.2. Vitamin D-Related Single Nucleotide Polymorphisms Analyses

Whole blood was collected in EDTA tubes and genomic DNA was obtained from blood samples (MagnaPure Compact, Roche, Monza, Italy); allelic discrimination was assessed through the real-time polymerase chain reaction system (LightCycler 480, Roche, Monza, Italy). The investigated gene SNPs were VDR (encoding VD receptor) rs7975232 (ApaI) C>A, rs731236 (TaqI) T>C, rs10735810 (FokI) T>C, rs11568820 (Cdx2) A>G, and rs1544410 (BsmI) G>A, CYP27B1 (encoding cytochrome 27B1 enzyme responsible for the active metabolite 1,25-VD production) rs4646536 (+2838) C>T and rs10877012 (-1260) G>T, CYP24A1 (encoding cytochrome 27B1 enzyme responsible for VD inactive metabolite 24,25-dyhydroxyvitamin D (24,25-VD) production) rs2248359 (3999) T>C, rs927650 (22776) C>T, and rs2585428 (8620) A>G, and finally GC (encoding VD transporter, VDBP) rs7041 (1296) A>C.

### 4.3. Statistical Analysis

All variables were tested for normality through the Shapiro–Wilk test. Normal variables were presented as the average and standard deviation, whereas non-normal variables were median values and the interquartile range (IQR) and categorical variables were numbers and percentages. Allele frequencies were analyzed for Hardy–Weinberg equilibrium. Kruskal–Wallis and Mann–Whitney tests were adopted for differences in continuous variables between genetic groups, considering a statistical significance with a two-sided *p*-value < 0.05. Stepwise multivariate logistic regression analysis was then performed including variables with a *p*-value below 0.05 in univariate analysis to evaluate factors able to predict clinical remission at three and twelve months of therapy (IBM Statistics 27.0 for Windows, Chicago, Illinois, USA).

Since no previous studies have analyzed the ability of VD genetics to predict clinical response to ADA in patients with CD, a prior calculation of the power of the study was not possible.

## 5. Conclusions

In conclusion, this is the first study investigating the role of VD-associated genes in evaluating the clinical remission at three and twelve months of therapy with ADA in patients with CD. The novelty of our results is the most important strength of the present study. However, further studies are required in order to confirm these preliminary data and to evaluate their potential role in clinical management.

## Figures and Tables

**Table 1:** Characteristics of patients. Categorical variables were described as numbers and frequencies, and linear ones as median and interquartile range (IQR).

Characteristics	Values
Number of patients	63
Age (year), median [IQR]	42 [32;55]
Male sex, n (%)	36 (57.1)
Perianal Disease, *n* (%)	10 (15.9)
Smoke (0 = no, 1 = smoker, 2 = ex), *n* (%)	31 (49.2)
	11 (17.5)
	21 (33)
Alcohol, n (%)	2 (3.2)
Intestinal localization, *n* (%)	L1 24 (38.1)
	L2 9 (14.3)
	L3 29 (46)
	L4 1 (1.6)
Surgery, n (%)	39 (61.9)
Comorbidities, n (%)	23 (36.5)
VD Supplementation, n (%)	31 (49.2)
Weight, median [IQR]	61 [57.5–70.5]
Height, median [IQR]	170 [164–174.5]
Years of disease, median [IQR]	13 [6.5–18.5]
Hb t0, median [IQR]	13.9 [12.58–14.56]
Calprotectin t0, median [IQR]	322.5 [130.5–657.5]
CRP > 1 t0, n (%)	34 (54.1)
Clinical response 3 months, *n* (%)	46 (73)
Clinical response 12 months, *n* (%)	40 (63.5)
Remission 3 months, *n* (%)	37 (58.7)
Remission 12 months, *n* (%)	36 (57.1)

**Table 2:** Median levels of calprotectin and hemoglobin according to vitamin D-related gene polymorphisms at the three different time points.

	*CYP27B1-1260 GG/GT*	*CYP27B1-1260 TT*	*CYP27B1 + 2838 CC*	*CYP27B1 + 2838 CT/TT*	*VDR ApaI CC*	*VDR ApaI CA/AA*	*VDR Cdx2 AA*	*VDR Cdx2 AG/GG*	*GC 1296 AA/AC*	*GC 1296 CC*	*CYP24A1 8620 AA/AG*	*CYP24A1 8620 GG*
***CALPROTECTIN ug/g (BL)***	301 (117–534)	1082 (996–/)	1064 (784–3608)	503 (300–1300)	798 (321–1829)	298 (92–511)	115 (74–196)	330 (163–830)	405 (213–997)	170 (53–500)		
***CALPROTECTIN ug/g (T3)***	49 (17–138)	1001 (129–/)	359 (70–1670)	49 (15–133)	224 (55–898)	42 (15–131)						
***CALPROTECTIN (T12)***	41 (15–237)	1585 (683–/)										
***HEMOGLOBIN g/dL (BL)***	13.90 (12.63–14.98)	10.70 (10.5–/)									13.50 (12.48–14.43)	14.9 (13.50–15.80)
***HEMOGLOBIN g/dL (T3)***	14.15 (13.08–15.03)	11.70 (10.30–/)									13.65 (12.33–14.88)	14.9 (13.65–15.90)
***HEMOGLOBIN g/dL (T12)***	14.30 (13.00–15.05)	11.20 (9.70–/)							13.70 (12.65–14.70)	14.50 (13.73–15.60)		

**Table 3:** Logistic regression analysis at three months of therapy. NSC: Not statistically comparable, as one group is missing; OR: *Odds ratio*, 95% IC: Interval of confidence at 95%.

	Remission after 3 Months of Therapy			
	Univariate		Multivariate	
	*p*-Value	OR (95% IC)	*p*-Value	OR (95% IC)
Age [≥50]	0.621	0.768 (0.269–2.190)		
Sex	0.941	0.963 (0.349–2.653)		
Weight [≥70 Kg]	0.889	1.08 (0.367–3.182)		
Smoke	0.023	0.506 (0.281–0.909)	0.035	0.480 (0.243–0.948)
Alcohol	NSC			
Years of disease >10	0.367	0.605 (0.203–1.804)		
Perianal Disease	0.543	0.656 (0.169–2.547)		
Surgery	0.634	0.776 (0.274–2.199)		
Systemic steroids	0.002	0.103 (0.025–0.422)	0.001	0.081 (0.018–0.366)
Mesar	0.808	0.871 (0.285–2.661)		
Topical steroids	0.386	0.438 (0.068–2.828)		
Immunosuppression	0.213	2.464 (0.597–10.178)		
Ab	0.434	1.789 (0.416–7.684)		
VD Supplementation	0.685	1.231 (0.415–3.364)		
*VDR* BsmI AA	NSC		NSC	
*GC 1296 CA/AA*	0.049	3.229 (0.994–10.491)		

**Table 4:** Logistic regression analysis at twelve months of therapy. NSC: Not statistically comparable, as one group is missing; OR: *Odds ratio*, 95% IC: Interval of confidence at 95%.

	Remission after 12 Months of Therapy			
	Univariate		Multivariate	
	*p*-Value	OR (95% IC)	*p*-Value	OR (95% IC)
Age [>50]	0.03	0.267 (0.081–0.883)		
Sex	0.248	1.964 (0.624–6.179)		
Weight [>70 Kg]	0.836	0.880 (0.263–2.950)		
Smoke	0.279	0.702 (0.370–1.332)		
Alcohol	NSC			
>10 aa mal	0.017	0.140 (0.028–0.698)		
Mal xian	0.072	0.236 (0.049–1.135)		
Surgery	0.691	0.786 (0.240–2.575)		
Systemic steroids	0.621	0.700 (0.170–2.882)		
Mesar	0.521	0.649 (0.174–2.426)		
Topical steroids	0.104	0.143 (0.014–1.487)		
Immunosuppression	0.331	2.286 (0.432–12.103)		
Ab	0.444	1.931 (0.358–10.422)		
VD Supplementation	0.004	0.143 (0.039–0.529)	0.006	0.050 (0.006–0.428)
CYP24A1 22776 TT	NSC			
VDR ApaI CA/AA	NSC			
VDR BsmI AA	NSC			
GC 1296 CA/AA	0.001	10 (2.479–40.331)	0.003	29.285 (3.160–271.367)

## Data Availability

Data is contained within the article.
